# Characterization of Polysaccharides Derived From *Cinnamomum cassia* and Their Potential of Antioxidant Activity and Protective Action Against Methomyl Induced Rats Damage With In Silico Docking

**DOI:** 10.1002/cbdv.202503347

**Published:** 2026-01-21

**Authors:** Nozza Bouzenna, Mabrouk Horchani, Sabah Dhibi, Hafsia Bouzenna, Fatma Guesmi, Anouar Feriani, Hichem Ben Jannet, Sirine Choura, Angelo Maria Giuffrè, Mohamed Chamkha, Najla Hfaiedh

**Affiliations:** ^1^ Faculty of Sciences of Gafsa, Laboratory of Biotechnology and Biomonitoring of the Environment and Oasis Ecosystems (LR21ES26) University of Gafsa Gafsa Tunisia; ^2^ Faculty of Sciences of Monastir Laboratory of Heterocyclic Chemistry Natural Products and Reactivity (LR11Es39) Medicinal Chemistry and Natural Products University of Monastir Monastir Tunisia; ^3^ Faculty of Sciences of Bizerte Laboratory of Risks Related to Environmental Stresses: Fight and Prevention Unit UR03ES06 University of Carthage Carthage Tunisia; ^4^ Laboratory of Environmental Bioprocesses Centre of Biotechnology of Sfax University of Sfax Sfax Tunisia; ^5^ Department of AGRARIA University Mediterranea of Reggio Calabria Reggio Calabria Italy

**Keywords:** characterization, *Cinnamomum cassia* polysaccharides, docking, methomyl, oxidative stress

## Abstract

Herbal medicine is an important field focused on the treatment and prevention of various health conditions that may occur due to oxidative stress. Our investigation aimed to determine the chemical composition, antioxidant, and the protective activity of polysaccharides isolated from *Cinnamomum cassia* (Cc‐Ps). Cc‐Ps underwent thorough characterization through various analytical methods, including UV, Fourier Transform Infrared (FTIR) spectroscopy, and HPLC. Our analysis showed that the Cc‐Ps exhibited highest amount of uronic acid. In addition, Cc‐Ps extract showed high ability to scavenge 2,2 diphenylpicrylhydrazyl radical (DPPH^●^), 2,2′‐azinobis‐(3 ethylbenzothiazoline‐6 sulfonic acid radical (ABTS^•+^), and NO free radicals in reaction system. There was a noticeable decrease in the activity of key antioxidant enzymes such a superoxide dismutase and glutathione (GSH) peroxidase accompanied by an increase in malondialdehyde, a marker of oxidative stress. MET led to a clear impairment of the histopathological structure of both the liver and kidneys. Pretreatment with the polysaccharide extract normalized all biochemical and oxidative parameters. Virtual screening using docking simulations against “*Drosophila melanogaster* Acetylcholinesterase” was conducted to assess the bio‐insecticidal properties of specific polysaccharide compounds derived from *C. cassia*. Polysaccharides were effectively protective in the liver and kidneys against the toxic effects of MET under our experimental conditions.

## Graphical abstract

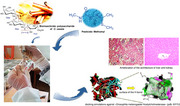

**Abbreviations**
ABTS: 2,2′‐azino‐bis(3‐ethylbenzothiazoline‐6‐sulfonic acid)
Akt: protein kinase B, PKB
EDTA: diaminotetracarboxylic acid
FoxO_1_
: Forkhead box O transcription factor
FRAP: ferric reducing antioxidant power
KH_2_PO_4_
: potassium dihydrogen phosphate
KOH: potassium hydroxide
Phe371: phenylalanin 371
Trp472: tryptophane 472
Trp83: tryptophane 83
Tyr374: tyrosine 374

## 1 Introduction

Methomyl (C_5_H_10_N_2_O_2_S), *S*‐methyl‐1‐*N*‐[(methyl carbamoyl)‐oxy]‐thioacetimidate (MET), is a carbamate pesticide used in multiple agricultural countries because of its highly effective biological activity for pest control and crop protection [1]. It is a broad‐spectrum carbamate pesticide that is available in both liquid and solid formulations, under the trade name: Lannate and is commonly used for various agricultural applications to control a wide range of pests. It inhibits the activity of acetylcholinesterase (AChE), a cholinergic enzyme that plays a crucial role in neurotransmission by breaking down acetylcholine, a key neurotransmitter in the body. It hydrolyzes acetylcholine into two components: acetic acid and choline. This process is essential for terminating the signal transmission between nerve cells, ensuring that nerve impulses are regulated and preventing excessive stimulation of receptors. The inhibitory action of MET leads to cholinergic overstimulation and neuromuscular dysfunction and can lead to a range of health risks, particularly affecting nerve function, muscle performance, and reproductive health and causes various health risks related to nerve, muscle reproductive functions [2].

In mammals, MET undergoes a metabolic pathway involving its conjugation with glutathione (GSH), resulting in the formation of a mercapturic acid derivative (MAD). This process takes place through the replacement of the *S*‐methyl group, allowing for the elimination of MAD by the liver and kidneys [3]. If MAD is not effectively cleared from the bloodstream, it can lead to nephrotoxicity. Furthermore, MET can also be hydrolyzed, producing *S*‐methyl‐*N*‐hydroxythioacetimidate. Once formed, MAD is rapidly broken down into carbon dioxide in the blood. An increase in carbon dioxide production can trigger hypoxic respiratory failure, highlighting the importance of efficient metabolic processing [4]. Carbamate compounds are known to induce oxidative stress in rat tissues by forming free radicals. Free radicals are significant factors in the toxicity of pesticides, as they contribute to the harmful effects on living organisms by stimulating the reduction of antioxidants or changes the enzyme systems that scavenge oxygen free radicals and the augmentation lipid peroxydation [4]. In addition, chronic exposure to methomyl causes hepatotoxicity, testicular damage, cytotoxicity, and suppression of brain activity [5].

Spices have been utilized in therapeutic contexts for the treatment of various ailments, primarily because of their potential functional properties. These natural ingredients are known for their beneficial effects on health and wellness, making them a valuable addition to holistic and alternative medicine practices [6]. Cinnamon is an indigenous spice that belongs to the laurel family (Lauraceae) and is primarily native to parts of Asia and Australia. This means that there are several types of cinnamon sold commercially around the world. One of the most important is *Cinnamomum cassia* or Cassia bark, which is native to southeastern China [7]. The beneficial properties of cinnamon are linked to the antioxidant effects of its bioactive compounds, including polyphenols, flavonoids, and polysaccharides. These compounds contribute to cinnamon's overall health benefits by combating oxidative stress in the body.

Polysaccharides play a crucial role as important biomacromolecules in all living organisms. Furthermore, numerous studies have indicated that polysaccharides derived from the spice *C. cassia* demonstrate a broad spectrum of pharmacological effects, including hypolipidemic, antitumor, antidiabetic, anti‐obesity, antimicrobial, hepato‐protective, immuno‐restorative, immunoregulatory, and anti‐inflammatory properties [8]. Polysaccharides may also be excellent sources of antioxidants [9]. However, to our knowledge, little information is available on the protective potential of Cinnamon polysaccharides compounds against methomyl‐induced toxicity in rats.

In this line of evidence, we characterize the polysaccharides derived from *C. cassia*. In addition, the in vitro antioxidant and protective potential of these biological macromolecules against methomyl‐induced toxicity in liver and kidney tissues were also investigated.

## 2 Materials and Methods

### 2.1 Chemicals and Reagents

Lannate (MET) 90% SP, the commercial formulation of MET [*S*‐methyl *N*‐(methyl carbamoyl oxy) thioacetimidate; C_5_H_10_N_2_O_2_S], was obtained from an agricultural products company. The reagents used for biochemical measurement of liver enzymes, kidney functions, and oxidative status, including quercetin, methanol, catechin, ethanol, butylated hydroxy toluene (BHT), potassium ferricyanide, gallic acid, 2‐thiobarbituric acid, sodium hydroxide, aluminium trichloride, hydroxyde of sodium, ferric chloride, and sodium phosphate buffer, were purchased from Sigma Aldrich, Mo, USA. Commercials Kit obtained from Spinreact, Spin.

### 2.2 Plant Material

The plant material used in this study consisted of dried bark of *C. cassia* (L.) J. Presl (Chinese cinnamon), purchased from the local herb market at Gafsa, Tunisia. It was identified in the National Institute of the Research in Rural Engineering, Waters, and Forest of Gabes, Tunisia [10]. The botanical identification was confirmed by a plant taxonomist from the Faculty of Sciences of Gafsa, FSG (Gafsa, Tunisia) using standard morphological and taxonomic keys and authoritative taxonomic databases. The samples were washed with tap water to remove sand and other impurities; then, they were dried at room temperature in the dark for 5 days and then in an oven at 38°C for 2 days. After that, the dried samples were crushed and stored in sterile boxes until use.

### 2.3 In Vitro Study

#### 2.3.1 Preparation of Polysaccharides Extract From *C. cassia*

The dried samples of *C. cassia* were powdered in a mill. Then, the ethanol (92%) was used to remove the liposoluble compositions from crude polysaccharides. The *C. cassia* polysaccharides were extracted using hot deionized water for 2 h. After extraction, this homogenate was collected and concentrated in a rotary evaporator at 55°C. Additionally, the protein fraction was removed from crude polysaccharides using Sevag reagent. Finally, the crude polysaccharides were obtained. The polysaccharide yields were determined according to the following equation:

$$\mathrm{Cc}-\mathrm{Ps}\;\mathrm{yield}\;\left(\%\right)=\frac{\mathrm{Weight}\;\mathrm{of}\;\mathrm{dried}\;\mathrm{crude}\;\mathrm{extraction}\;\left(g\right)}{\mathrm{Weight}\;\mathrm{of}\;\mathrm{each}\;\mathrm{powder}\;\mathrm{sample}\;\left(g\right)}\times 100$$

### 2.4 Physicochemical Characterization of Polysaccharides Isolated From *C. cassia* (Cc‐PS)

#### 2.4.1 Colorimetric Assays

The phenol‐sulfuric acid method was used to determine the carbohydrate content of Cc‐PS. d‐glucose was used as standard [11]. The uronic acid content was estimated by the carbazole method using galacturonic acid as standard [12]. The protein content in Cc‐PS was determined by the colorimetric method developed by [13].

#### 2.4.2 Ultraviolet–Visible Spectroscopy (UV–Vis)

UV–Vis absorption spectra of Cc‐PS were performed by a UV–vis spectrophotometer (Agilent Technologies Cary 60 UV–Vis) between 200 and 400 nm. A concentration of Cc‐PS (1 mg/mL) was prepared using distilled water as solvent. Distilled water was used as a reference, and nucleic acids and proteins were measured at 280 and 260 nm.

#### 2.4.3 Fourier Transform Infrared Spectroscopy (FTIR)

The infrared spectrum of Cc‐PS is obtained using an FTIR spectrophotometer (Perki‐Elmer spectrum 100 FTIR Spectrometer) equipped with an ATR A225 diamante. In this case, 2 mg of the sample deposited on an ATR was analyzed directly, and measurements were taken in a frequency range from 4000 to 500 cm^−1^. Data were analyzed using OriginPro 8 software.

#### 2.4.4 Analysis of Monosaccharide Composition by HPLC

The monosaccharide composition of Cc‐Ps was performed using the HPLC method described by [14]. Briefly, 20 mg of Cc‐PS were hydrolyzed to prepare monosaccharides using sulfuric acid (2 M) at 110°C for 8 h. Acetonitrile and phosphate buffer were used as the mobile phase. The wavelength of UV detection was set to be 250 nm.

#### 2.4.5 Antioxidant and Anti‐Inflammatory Activities Analysis of Cc‐PS

The antioxidant and anti‐inflammatory potentials of Cc‐PS were determined by radical scavenging assays: the 2,2 diphenylpicrylhydrazyl radical (DPPH^•^), the 2,2′‐azinobis‐(3 ethylbenzothiazoline‐6 sulfonic acid radical (ABTS^•+^), and nitrite test (NO^•^).

The ability of Cc‐Ps to scavenge DPPH free radicals was determined as described, previously by [15]. Briefly, a concentration of DPPH (0.1 mM) was prepared using ethanol as solvent. Then, 1 mL of Cc‐Ps at different concentrations (50–800 µg/mL) was mixed with 0.5 mL of DPPH reagent. The reaction mixture was incubated in a dark room for 30 min. The result was measured at 515 nm. At this wavelength, we unlock the potential to minimize interference from the sample's absorbance. By incorporating negative controls, by incubating the DPPH alone in the reaction medium, we pave the way for clarity and precision in our pursuit of knowledge without the antioxidant reagent. The results can be standardized and compared to the antioxidant, BHT, illuminating pathways for deeper insights.

The ability of Cc‐Ps to scavenge DPPH free radicals was calculated according to the following equation:

$$\%\;\mathrm{DPPH}\;\mathrm{scavenging}=\left[\frac{\left(A_{\mathrm{control}}-A_{\mathrm{test}}\right)}{A_{\mathrm{control}}}\right]\times 100$$

where *A*_control_ is the absorbance of the control reaction and *A*_test_ is the absorbance of the extract reaction.

Antioxidant activities of Cc‐Ps were also analyzed by investigating their ability to scavenge the ABTS^•+^ free radical using the method described by [16]. When combined with an oxidant (2.45 mM potassium persulfate), ABTS (7 mM in 20 mM sodium acetate buffer, pH 4.5) reacts to create a stable, dark blue–green radical solution following 12–16 h of incubation in the dark (4°C). The solution was then diluted to an absorbance of 0.7 ± 0.01 at 734 nm to form the test reagent. Reaction mixtures containing 20 µL of sample and 3 mL of reagent were incubated in a water bath at 30°C for 30 min. As unpaired electrons are sequestered by antioxidants in the sample, the test solution turns less colored, and the absorbance at 734 nm is reduced. The findings can be standardized and evaluated against a reference antioxidant like Trolox for comparison purposes. Radical scavenging activity was calculated using the formula:

$$\%\;\mathrm{Inhibition}=\left[\frac{\left(A_{\mathrm{control}}-A_{\mathrm{test}}\right)}{A_{\mathrm{control}}}\right]\times 100$$

where *A*_control_ is the absorbance of the control reaction and *A*_test_ is the absorbance of the extract reaction.

The nitric oxide activity of Cc‐Ps was determined according to the method of [17]. The reaction mixture containing sodium nitroprusside (2 mL, 10 mM in 0.5 M phosphate buffer solution, pH 7.4) and 250 µL of Cc‐Ps at different concentrations (50–800 µg/mL) was incubated at 25°C for 150 min. Then, for each reaction mixture, the aliquot part (0.5 m) was added to the test tube containing 1 mL sulfanilamide (1% prepared in 100 mL 5% phosphoric acid) and incubated at 25°C for 5 min. Then, 1 mL of (1‐napthyl)‐ethylenediamine (1%) was added to each mixture and incubated for 30 min at 25°C. Absorbance was measured at 546 nm. The ability of Cc‐Ps to scavenge NO free radicals was measured as the following formula:

$$\%\;\mathrm{Inhibition}=\left[\frac{\left(A_{\mathrm{control}}-A_{\mathrm{test}}\right)}{A_{\mathrm{control}}}\right]\times 100$$

where *A*_control_ is the absorbance of the control reaction and *A*_test_ is the absorbance of the extract reaction.

### 2.5 In Vivo Study

#### 2.5.1 Animals

In this study, 24 healthy male albino Wistar rats, each aged 6 months, were utilized as subjects. These rats were housed in polypropylene cages and provided with a nutritionally balanced diet alongside fresh tap water. They were kept under controlled room temperature conditions and maintained on a 12‐h light and dark cycle to simulate natural living conditions. The experimental procedures involving the rats received ethical approval from the Medical Ethical Committee for the Care and Use of Laboratory Animals at the Faculty of Sciences of Gafsa, Tunisia, ensuring compliance with established guidelines for the humane care and use of laboratory animals.

#### 2.5.2 Pesticide Preparation

The methomyl‐based pesticide, known as Lannate, was procured from a pharmacy. The concentration of methomyl pesticides utilized in this study was 200 g/L, based on findings reported by [18].

#### 2.5.3 Experimental Design

Three‐month‐old male Wistar rats, each weighing approximately ±90 g, were maintained in a breeding facility at 22°C, under constant photoperiod conditions. These rats were divided into four groups, each consisting of six rats:

**Group T**: It served as the control group.
**Group P**: This group received 100 mg/kg/day of the polysaccharides extract orally by gavage throughout the treatment period.
**Group MET**: It was administered 4 mg/kg/day of methomyl (MET) via gavage for the subsequent 3 days.
**Group P + MET**: It was pretreated with polysaccharides for 17 days and then treated with methomyl for the last 3 days, at a dosage of 4 mg/kg/day through gavage.

#### 2.5.4 Organ's Collection

The animals were weighed daily, and after 17 days of treatment, they were sacrificed via cervical disruption. The liver and kidneys were swiftly excised and weighed; one portion was frozen at −80°C for analysis, whereas another was fixed in formalin for immediate histopathological assessment. Blood was centrifuged, and serum was preserved in a deep freeze (−20°C) until further analysis.

### 2.6 Biochemical Assays

#### 2.6.1 Assessment of Serum Markers

Serum concentrations of glucose, urea, creatinine, and activities of aspartate transaminase (AST) were measured using kit methods (Spinreact).

**Protein content in tissue extracts**: The determination was conducted using the Lowry method [13], employing bovine serum albumin as the reference standard for comparison.

### 2.7 Lipid Peroxidation (LPO) Levels

LPO levels can be assessed using the thiobarbituric acid reactive substances (TBARS) method, which was developed by Yagi [19]. In this assay, 125 µL of supernatant (S1) obtained from kidney and liver samples is combined with 175 µL of a 20% trichloroacetic acid solution that includes 1% butyl‐hydroxytoluene. The mixture is then centrifuged at 1000 × *g* for 10 min at 4°C to separate the components. Next, 200 µL of the resulting supernatant (S2) is mixed with 40 µL of 0.6 M hydrochloric acid and 160 µL of a 0.72 mM thiobarbituric acid solution. This mixture is heated at 80°C for 10 min to facilitate the reaction. The absorbance of the solution is measured at 530 nm. TBARS levels are then calculated using an extinction coefficient of 156 mM^−1^/cm and are expressed in nanomoles per milligram of protein.

### 2.8 Catalase Activity (CAT)

The enzymatic activity was evaluated following the methodology established by Aebi [20]. In this process, a reaction mixture consisting of 1 mL was prepared using 100 mM phosphate buffer (pH 7), 100 mM hydrogen peroxide (H_2_O_2_), and 20 µL of kidney or liver homogenate (which corresponds to approximately 1–1.5 mg of protein). The breakdown of H_2_O_2_ was monitored at a temperature of 25°C by measuring the decrease in absorbance at 240 nm over one minute. The enzyme activity was then calculated employing an extinction coefficient of 0.043 mM^1^/cm and expressed in international units (I.U.), representing the micromoles of H_2_O_2_ decomposed per minute per mg of protein.

### 2.9 The Superoxide‐Dismutase (SOD) Activity

The evaluation method involves assessing the ability to inhibit the photoreduction of nitroblue tetrazolium (NBT), as described by Durak [21]. A single unit of SOD is defined as the amount that reduces NBT photoreduction by 50%. The activity of SOD is expressed in terms of units per milligram of protein at a temperature of 25°C.

### 2.10 GSH‐Peroxidase (GPX) Activity

The analysis was conducted following the methodology established by Flohe and Gunzler [22]. The enzymatic activity, measured at a temperature of 25°C, was expressed as micromoles of GSH oxidized per minute per gram of protein.

### 2.11 Molecular Docking Procedure

Molecular docking simulations were performed via Auto Dock 4.2 program package [23]. The crystal structure of “AChE from *Drosophila melanogaster* complex with tacrine derivative 9‐(3‐iodobenzylamino)‐1,2,3,4‐tetrahydroacridine” [24] was downloaded from the RSCB protein data bank. The preparation of the receptor input file involved the removal of water molecules, followed by the addition of missing hydrogen atoms and Gasteiger charges to the system. Subsequently, AutoDock Tools were utilized to prepare all ligand and protein files in PDBQT format. To optimize efficiency during docking, grid maps were pre‐calculated using Auto Grid. Additionally, the geometries of all compounds were optimized using ACD 3D Viewer software, whereas Discovery Studio 2017R2 was employed for the visualization and analysis of molecular interactions.

### 2.12 Histopathological Examination

For the histopathological evaluation, formalin‐fixed liver and kidney tissues were routinely processed. They were embedded in paraffin and then sectioned into slices measuring 3–4 µm. The sections were stained using hematoxylin and eosin (H&E), a common staining technique that highlights cellular details.

### 2.13 Statistical Analysis

All experimental measurements were conducted in triplicate to ensure accuracy and reliability, and the results are presented as the mean ± three standard deviations [mean (SE) ± SD]. To evaluate the data statistically, a one‐way analysis of variance (ANOVA) was performed. A significance level of *p *≤ 0.05 was used to determine statistical significance.

## 3 Results

### 3.1 Colorimetric Assays

The neutral sugars, uronic acids, and proteins of the polysaccharide extracted from the *C. cassia* are listed in Table 1. The yield of Cc‐Ps is amount 6.75% and the amount of neutral sugars is 78.56%. In addition, the amount of uronic acids is about 17.45%.

**TABLE 1:** Chemical characterization of polysaccharides isolated from *Cinnamomum cassia* (Cc‐Ps).

Chemical composition	^a^Value (%)
Yield	6.75 ± 0.46
Sugars neutral	78.56 ± 3.21
Uronic acids	17.45 ± 1.25
Proteins	ND^b^

*Note*: Values are expressed as mean ± standard deviation (*n* = 3).

^a^Percentage by weight of lyophilisate (Cc‐Ps).

^b^Non detected.

### 3.2 UV–Vis Spectroscopy

**FIGURE 1:**
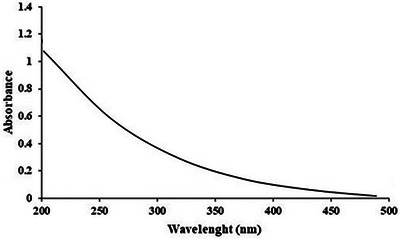
UV analysis of Cc‐Ps extract.

Our analysis showed the absence of peaks in the 260–280 nm range (Figure 1), confirming that Cc‐Ps extract is a pure product without nucleic acid or protein. This result confirmed the purification steps applied in this study.

### 3.3 FTIR Spectroscopy

**FIGURE 2:**
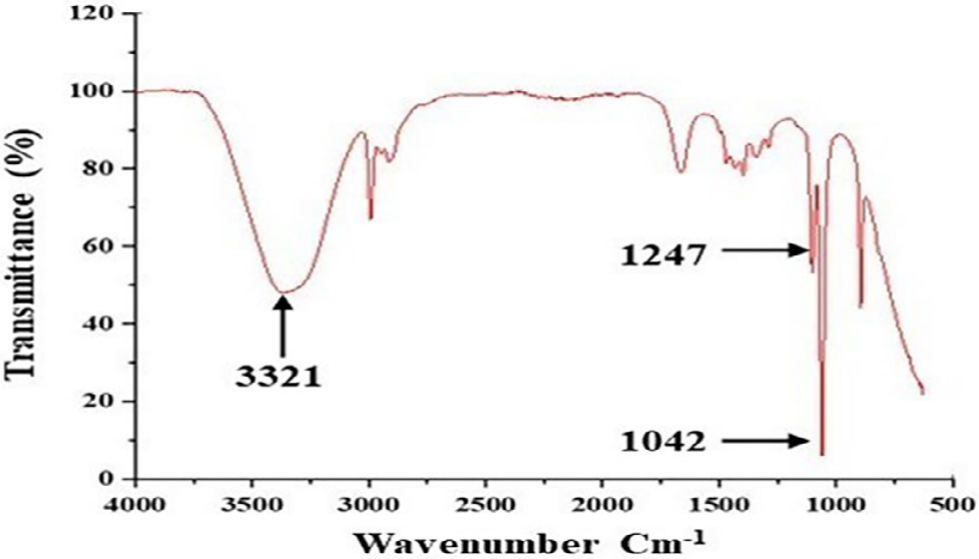
FTIR spectra of Cc‐PS.

The functional groups of Cc‐Ps were performed by FTIR analysis (Figure 2). The FTIR spectrums of Cc‐Ps have the same polysaccharide absorption bands, with a strong, broad absorption bond observed at 3321 cm^−1^, associated with hydroxyl (─OH) groups of galacturonic acids. In addition, the absorption band observed at 1248 cm^−1^ was also attributed to the trace of uronic acids. A strong absorption observed at around 1039 cm^−1^ presented the stretching vibration of the C─O─H side groups and the vibration of the C─O─C glycosidic bond.

### 3.4 HPLC Analysis of Cc‐Ps

**FIGURE 3:**
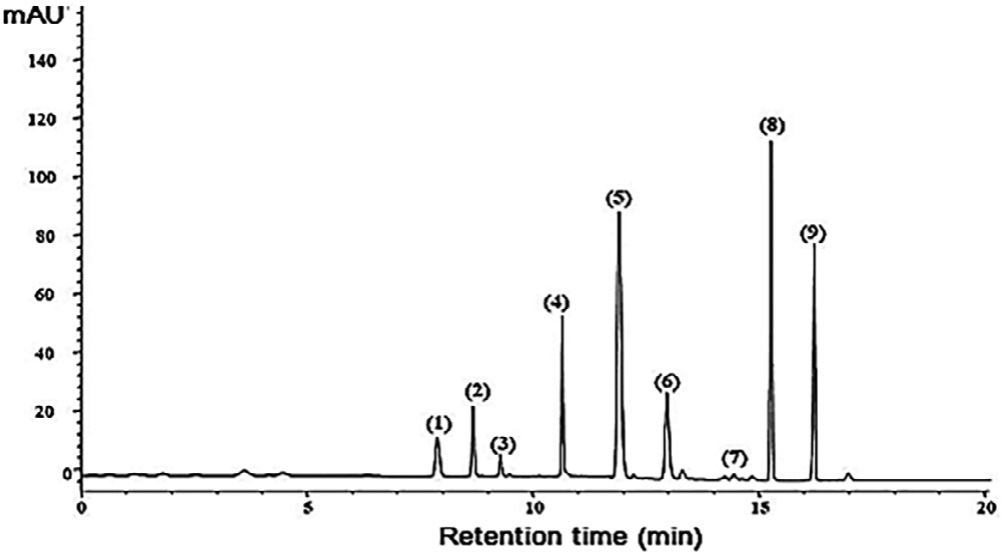
HPLC analysis of Cc‐Ps. Peaks: 1: mannose; 2: ribose; 3: rhamnose; 4: glucuronic acid; 5: galacturonic acid; 6: glucose; 7: xylose; 8: galactose; 9: arabinose.

The monosaccharides compositions of the Cc‐Ps were performed by HPLC analysis, and the results are listed in Table 2 and Figure 3. Major monosaccharides detected in Cc‐Ps are galactose, arabinose, and glucose. In our analysis, nine monosaccharides were identified in Cc‐Ps. Mannose, ribose, rhamnose, glucuronic acid, galacturonic acid, glucose, xylose, galactose, and arabinose found in this polymer. Galactose was the major components (25.32%) followed by arabinose (22.63%) and glucose (17.52%), and minor monosaccharides including xylose and rhamnose.

**TABLE 2:** Monisaccharides composition of polysaccharides isolated from *Cinnamomum cassia* (Cc‐Ps) by HPLC analysis.

Short name	Sugar components (%)
(1) Mannose	4.63
(2) Ribose	5.63
(3) Rhamnose	2.41
(4) d‐glucuronic acid	7.52
(5) Galacturonic acid	8.63
(6) Glucose	17.52
(7) Xylose	1.85
(8) Galactose	25.32
(9) Arabinose	22.63

### 3.5 In Vitro Evaluation of the Antioxidant Potential of Cc‐Ps

#### 3.5.1 DPPH‐Free Radical Scavenging

**FIGURE 4:**
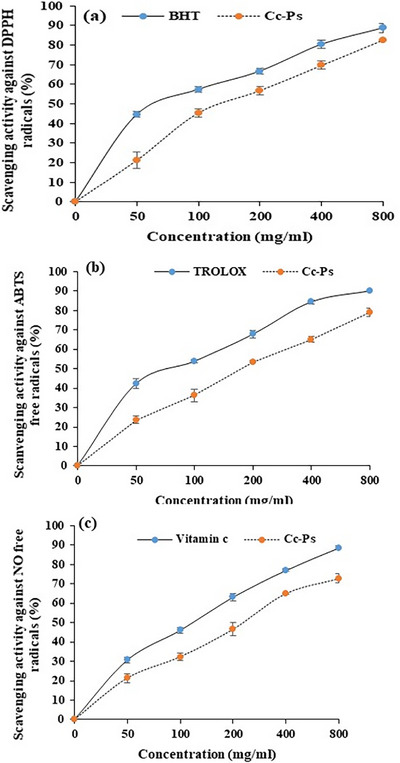
Antioxidant capacity of Cc‐Ps against the DPPH (a), ABTS^−+^ (b), and NO radicals (c). BHT, TROLOX and vitamin C were used as the standard, respectively. All the experiments were done in triplicate. BHT, butylated hydroxy toluene; Cc‐Ps, polysaccharides isolated from *Cinnamomum cassia*.

The capacity of Cc‐Ps to neutralize DPPH‐free radicals in reaction system at different concentrations is listen in Figure 4a. Cc‐Ps had a strong ability to scavenge DPPH free radicals compared with the standard (BHT). The percentage of inhibition varied between 22.21 at 50 µg/mL and 82.7 at 800 µg/mL. Our analysis showed that this polymer exhibited a strong anti‐free radical activity against the DPPH radicals.

#### 3.5.2 ABTS or TEAC Test

Figure 4b shows the scavenging capacity of Cc‐Ps against ABTS^−+^ free radical. The inhibition rate of Cc‐Ps varied between 23.52% at 50 µg/mL and 79% at 800 µg/mL. This result shows that the polymer has an effective capacity to neutralize ABTS‐free radicals in the reaction system.

#### 3.5.3 Anti‐Inflammatory Activity Using the Nitrite Test

The ability of Cc‐Ps to neutralize NO^•^ free‐radical is summarizing in Figure 4c. In this analysis the inhibition rate of this free radical increases with increasing concentration, for both vitamin C and Cc‐Ps. The ability of Cc‐Ps to neutralize NO free‐radicals ranged from 21.32% to 72.17%.

### 3.6 Protective Effect of Cc‐Ps Against Methomyl Toxicity

#### 3.6.1 Biochemical Parameters

Table 3 shows biochemical parameters related to liver and kidney functions. Generally, the groups of rats allowed to drink fluids free of MET (Cc‐Ps, T) showed results of insignificant differences. The activity of AST in the control rats (T) was recorded at 201.6 U/L, but for rats treated with MET, the activity was recorded at 347.6 U/L, achieving highly significant differences (*p *≤ 0.01). The group pretreated with Cc‐Ps and then given MET (Cc‐Ps + MET) recorded AST activities very close to that of control results.

**TABLE 3:** Some biochemical parameters of liver enzymes and kidney functions in male rats treated with methomyl, with and without polysaccharide extract, and its mixture with methomyl.

	Control (T)	Cc‐Ps	MET	Cc‐Ps + MET
Glu (mmol/L)	4.25 ± 0.6	4.04 ± 0.52^###^	8.09 ± 0.61***	4.69 ± 0.8^##^
AST (U/L)	201.61 ± 23.3	272.2 ± 35.25^###^	347.6 ± 34.83***	259.81 ± 49.41^##^
Urea (mmol/L)	4.46 ± 0.88	4.42 ± 0.42^##^	5.8 ± 0.54**	4.56 ± 0.54^#^
Crea (µmol/L)	35.16 ± 0.82	35.14 ± 0.41^##^	42.54 ± 0.8***	35.25 ± 0.56^#^

*Note*: Values correspond to the mean of 6 measurements ± SD. Student test: *** (P ≤ 0.001), ** (P ≤ 0.01) indicate significant differences between Cc‐Ps and control rats (T). ^###^ (P ≤ 0.001), ^##^ (P ≤ 0.01), ^#^ (P ≤ 0.05) indicate significant difference between (Cc‐Ps and Cc‐Ps + MET) and (MET) rats.

Abbreviation: Cc‐Ps, polysaccharides isolated from Cinnamomum cassia.

A notable finding was that the serum levels of glucose were significantly elevated in the Cc‐Ps rats compared to the control group. In contrast, the rats that received pretreatment with the polysaccharides extract from *C. cassia* exhibited a significant reduction in glucose levels.

Significant elevation (*p *≤ 0.05) in urea concentrations was obtained in MET treatments (5.8 mmol/L) compared with control value (4.46 mmol/L). These effects were significantly decreased in the polysaccharide and MET‐treated rats with extract (Cc‐Ps + MET) (4.56 mmol/L) compared to the MET‐treated groups.

Highly significant elevation (*p *≤ 0.01) in creatinine concentrations was obtained in MET treatments and reached 42.54 µmol/L compared to control group (35.16 µmol/L). Co‐administration of the tested extracts restored creatinine concentration to the normal levels.

#### 3.6.2 Levels of LPO in Liver and Kidney Tissues

**FIGURE 5:**
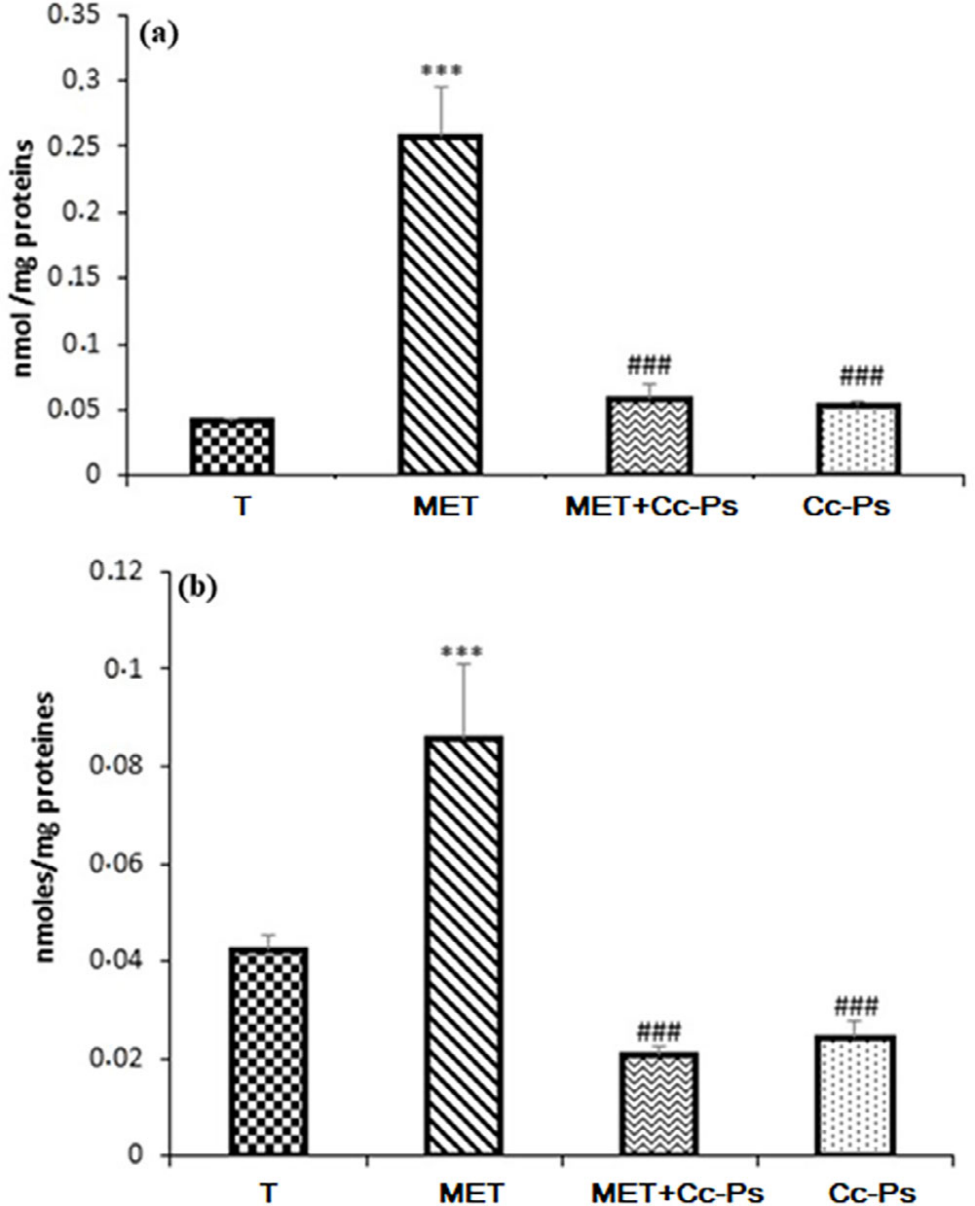
Levels of TBARS in the kidneys (a) and liver (b) of control rats (T), rats consuming polysaccharide (Cc‐Ps), rats treated with methomyl (MET), and rats pretreated with polysaccharide and then by methomyl (Cc‐Ps + MET). Values correspond to the mean of six measurements ± SD. Student test: *** (*p *≤ 0.01) indicates significant difference between (Cc‐Ps and Cc‐Ps + MET) and (MET) rats. All the experiments were done in triplicate.

The levels of LPO in liver and kidney tissues are summarized in Figure 5. TBARS recorded significant elevation (*p *≤ 0.01), reaching 0.25 nmol/mg protein and 0.08 nmol/mg protein in kidney and liver homogenates, respectively, in the rats treated only with MET. Such significant elevation was greatly limited (*p *≤ 0.01) by the pretreatment with Cc‐Ps extract.

#### 3.6.3 Changes of Antioxidant Enzyme Activities in Liver and Kidney Homogenates

**FIGURE 6:**
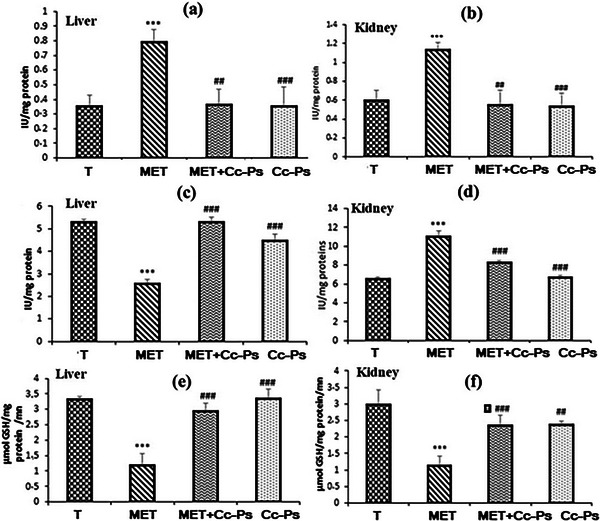
Levels of the activities of (a) and (b) catalase (CAT), (c) and (d) superoxide‐dismutase (SOD), and (e) and (f) glutathione peroxidase (GPx) and of control rats (T), rats consuming polysaccharide (Cc‐Ps), rats treated for the last 3 days with methomyl (MET), and rats pretreated with polysaccharide and then by methomyl (Cc‐Ps + MET). Values correspond to the mean of six measurements ± SD. Student test: *** (*p *≤ 0.01) indicates significant differences between (Cc‐Ps) and control rats (T). ### (*p* ≤ 0.01) indicates significant difference between (Cc‐Ps and Cc‐Ps + MET) and (MET) rats. All the experiments were done in triplicate.

MET treatment induced a highly significant increase in CAT activity in the liver and kidney tissues and a significant decrease in SOD and glutathione peroxidase (GPx) activities compared with control rats (Figure 6). However, the Cc‐Ps pretreatment (Cc‐Ps + MET) showed important changes in the activities of these enzymes which were close to normal.

### 3.7 Molecular Docking Study

#### 3.7.1 Insight Into the Receptor Used

The inhibition of AChE is recognized as the primary mechanism through which organophosphates exert their effects, as highlighted by the research of several structural biology and crystallography groups. AChE is a crucial enzyme responsible for the hydrolysis of the neurotransmitter acetylcholine, which is vital in regulating synaptic transmission across all animal species. This enzyme's role includes terminating the synaptic action of acetylcholine, thereby ensuring proper communication between nerve cells. The enzyme AChE is classified as a phase I metabolic enzyme and plays a significant role in metabolizing various internal and external substrates in pests. This group of metabolic enzymes encompasses a broad spectrum of enzymes that are capable of breaking down several chemical insecticides, including carbamates, organophosphates, and pyrethroids. The effectiveness of insecticides can be significantly impacted by alterations in the quantity of specific target enzymes. This highlights the necessity for the development of agents that utilize new and diverse mechanisms for insect control. In this context, AChE (pdb: 6XYU) has been identified as a promising target for assessing the bioinsecticidal activities of the primary phytocompounds found in the polysaccharides of *C. cassia*.

The objective of our research was to explore the interaction modes of receptor–ligand complexes, with a specific focus on identifying optimal orientations for ligand binding within the active site of the compound known as “tacrine derivative 9‐(3‐iodobenzylamino)‐1,2,3,4‐tetrahydroacridine.” This investigation aims to enhance our understanding of how ligands interact with receptors at a molecular level.

#### 3.7.2 The Docking Study Outcomes

**FIGURE 7:**
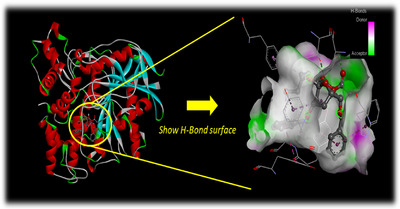
3D model (H‐bond surface) of the most effective ligand “(2*R*,3*R*,4*R*,5*S*,6*R*,8*S*,10*S*)‐5‐acetoxy‐10‐benzyloxy‐8‐benzyloxymethyl‐2‐ethyl‐3‐methyl‐1,7‐dioxaspiro[5.5]undecan‐4‐ol” in the binding cavity of “*Drosophila melanogaster* Acetylcholinesterase” (pdb: 6XYU).

**FIGURE 8:**
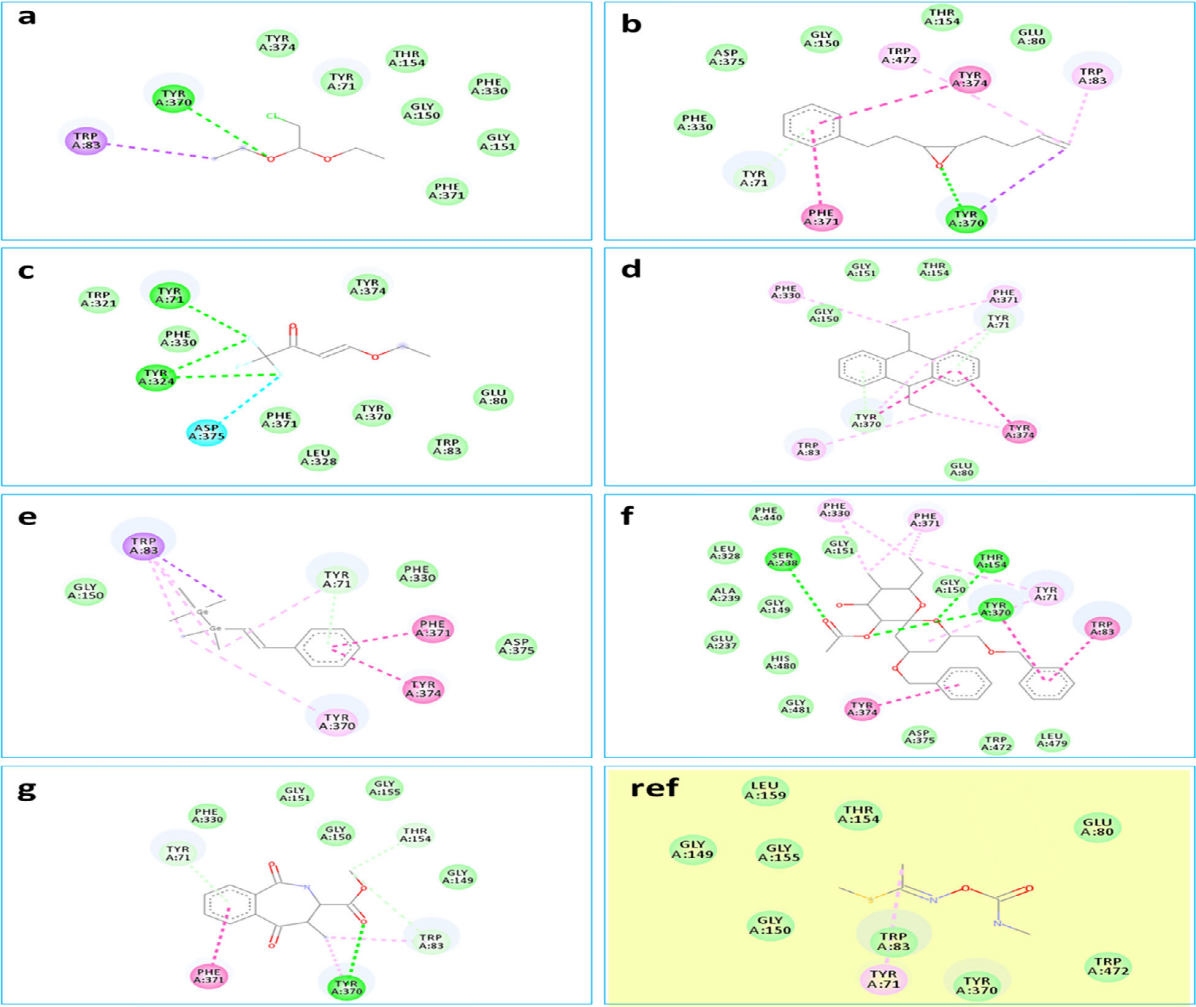
2D docking models of different interactions formed by the docked molecules: (a) 2‐chloro‐1,1‐diethoxy‐ethane, (b) *cis*‐3,4‐epoxy‐1‐phenyl‐7‐octene, (c) 4‐ethoxy‐1,1,1‐trifluoro‐3‐buten‐2‐one, (d) *trans*‐9,10‐diethyl‐9,10‐dihydroanthracene, (e) styrylpentamethyldigermane, (f) (2*R*,3*R*,4*R*,5*S*,6*R*,8*S*,10*S*)‐5‐acetoxy‐10‐benzyloxy‐8‐benzyloxymethyl‐2‐ethyl‐3‐methyl‐1,7‐dioxaspiro[5.5]undecan‐4‐ol, (g) methyl(3S*,4R*)‐2,3,4,5‐tetrahydro‐4‐methyl‐1,5‐dioxo‐1*H*‐benz[c]azepine‐3‐carboxylate and (ref) methomyl within the active site of “*Drosophila melanogaster* Acetylcholinesterase” (pdb: 6XYU).

As Table 4 displays, in the form of free‐binding energy values, the obtained results indicating the effects of the analyzed ligands toward the selected target enzyme compared to the methomyl show that all phytomolecules have significant docking score especially for(2*R*,3*R*,4*R*,5*S*,6*R*,8*S*,10*S*)‐5‐acetoxy‐10‐benzyloxy‐8‐benzyloxymethyl‐2‐ethyl‐3 methyl–1,7‐dioxaspiro [5.5] undecan‐ 4‐ol (−7.9 kcal/mol). This ligand was found to be the most active compound and Figure 7 shows by the H‐bond surface (green zone) the importance of the ester function and the “1,7‐dioxaspiro[5.5]undecan” fragment in the stability of the complex by the formation of intermolecular hydrogen bonds which contributes to inhibition of AChE. Figure 8f exhibits that this ligand is involved in many conventional hydrogen bonds with Thr154, Ser238, and Tyr370 in addition to several hydrophobic contacts as Alkyl/Pi–Alkyl with Tyr71, Phe330, Phe371, and Trp472 and Pi–Pi with Trp83, Tyr374. The tabulated data show that the second most effective ligands are styrylpentamethyldigermane and methyl(3S*,4R*)‐2,3,4,5‐tetrahydro‐4‐methyl‐1,5‐dioxo‐1*H*‐benz[c]azepine‐3‐carboxylate (−7.7 kcal/mol) which display some interesting interactions detailed in Figure 8e,g and Table 4.

**TABLE 4:** Docking results of the docked ligands with their binding energy scores and interacting residues within the binding cavity of “*Drosophila melanogaster* Acetylcholinesterase” (pdb: 6XYU).

Docked compounds	Interacting residues	Binding energy (kcal/mol)
2‐chloro‐1,1‐diethoxy‐ethane	Van der Waals: Tyr71, Gly150, Gly151, Thr154, Phe330, Phe371, Tyr374; H bond: Tyr370*; Pi–Sigma: Trp83	−4.7
*cis*‐3,4‐epoxy‐1‐phenyl‐7‐octene	Van der Waals: Glu80, Gly150, Thr154, Phe330, Asp375; H bond: Tyr370*; Alkyl/Pi–Alkyl: Trp83, Trp472; Pi–Pi: Phe371, Tyr374; Pi‐Donor Hydrogen bond: Tyr71; Pi–Sigma: Tyr370	−7.5
4‐ethoxy‐1,1,1‐trifluoro‐3‐buten‐2‐one	Van der Waals: Glu80, Trp83, Trp321, Leu328, Phe330, Tyr370, Phe371, Tyr374; H bond: Tyr71, Tyr324**; Halogen (Fluorine): Asp375	−5.6
*trans*‐9,10‐diethyl‐9,10‐dihydroanthracene	Van der Waals: Glu80, Gly150, Gly151, Thr154; Alkyl/Pi–Alkyl: Tyr71, Trp83, Phe330, Tyr370, Phe371, Tyr374; Pi–Pi: Tyr370, Tyr374; Pi‐donor hydrogen bond: Tyr71, Tyr370	−7.4
Styrylpentamethyldigermane	Van der Waals: Gly150, Phe330, Asp375; Alkyl/Pi–Alkyl: Tyr71, Trp83, Tyr370; Pi–Pi: Phe371, Tyr374; Pi‐donor hydrogen bond: Tyr71; Pi–Sigma: Trp83	−7.7
(2*R*,3*R*,4*R*,5*S*,6*R*,8*S*,10*S*)‐5‐acetoxy‐10‐benzyloxy‐8‐benzyloxymethyl‐2‐ethyl‐3‐methyl‐1,7‐dioxaspiro[5.5]undecan‐4‐ol	Van der Waals: Gly149, Gly150, Gly151, Glu237, Ala239, Leu328, Asp375, Phe440, Trp472, Leu472, His480, Gly481; H bond: Thr154*, Ser238*, Tyr370*; Alkyl/Pi–Alkyl: Tyr71, Phe330, Phe371, Trp472; Pi–Pi: Trp83, Tyr374	−7.9
Methyl(3S*,4R*)‐2,3,4,5‐tetrahydro‐4‐methyl‐1,5‐dioxo‐1*H*‐benz[c]azepine‐3‐carboxylate	Van der Waals: Gly149, Gly150, Gly151, Gly155, Phe330; H bond: Tyr370*; Alkyl/Pi–Alkyl: Trp83, Tyr370; Pi–Pi: Phe371; carbon hydrogen bond: Trp83, Thr154; Pi‐donor hydrogen bond: Tyr71	−7.7
Methomyl (reference)	Van der Waals: Glu80, Trp83, Gly149, Gly150, Thr154, Gly155, Leu159, Tyr370, Leu472; Pi–Alkyl: Tyr71	−4.3

On another hand, *cis*‐3,4‐epoxy‐1‐phenyl‐7‐octene was found to be the third most active ligand. Indeed, it displayed an H‐bond with Tyr370 via its epoxy pharmacophore besides to some other interactions as Alkyl/Pi–Alkyl with Trp83 and Trp472, Pi–Pi with Phe371 and Tyr374, Pi‐donor hydrogen bond with Tyr71 as well as Pi–Sigma with Tyr370 (Figure 8b). The fourth most effective ligand is *trans*‐9,10‐diethyl‐9,10‐dihydroanthracene, the inhibition capability of this compound is perceptible through only the hydrophobic interactions such as Alkyl, Pi–Alkyl, and Pi–Pi (Figure 8d), whereas 4‐ethoxy‐1,1,1‐trifluoro‐3‐buten‐2‐one can inhibit the target enzyme through three hydrogen bonds with Tyr71 and Tyr324 and halogen contact with Asp375 (Figure 8c). Finally, 2‐chloro‐1,1‐diethoxy‐ethane (−4.2 kcal/mol) was found to be the weakest ligand, showing only two interactions: an H bond with Tyr370 and a Pi–Sigma with Trp83 (Figure 8a).

### 3.8 Histopathological Changes

**FIGURE 9:**
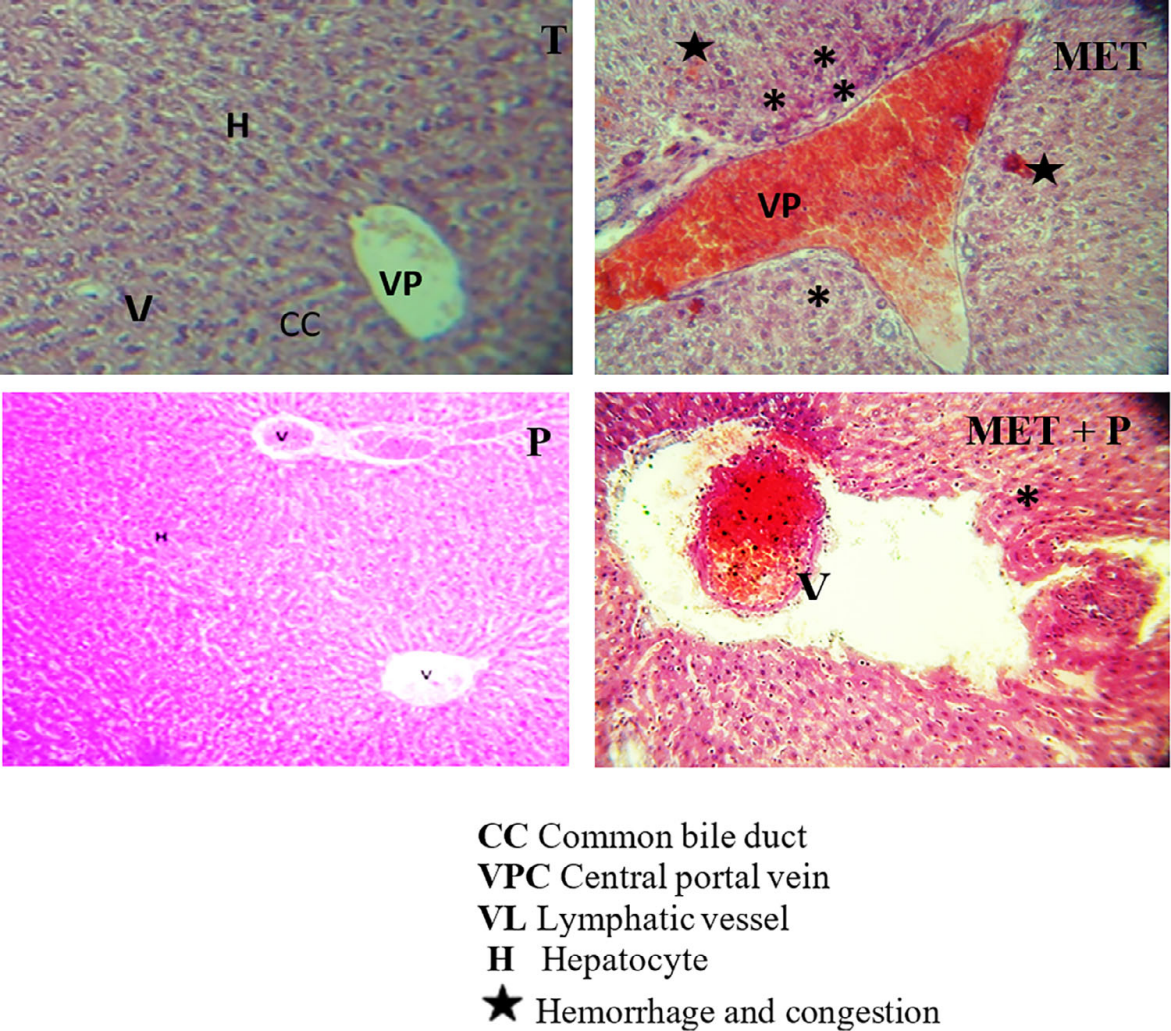
Liver histopathological changes induced by exposure to MET. Representative photographs showing (T) a control rat liver section, (Cc‐Ps) a liver section from a rat consuming polysaccharide extract from *Cinnamon cassia*, (MET) liver sections from methomyl‐treated rats, and (MET + Cc‐Ps) liver sections from polysaccharide pretreated rats. Hematoxylin/eosin staining; magnification ×200.

**FIGURE 10:**
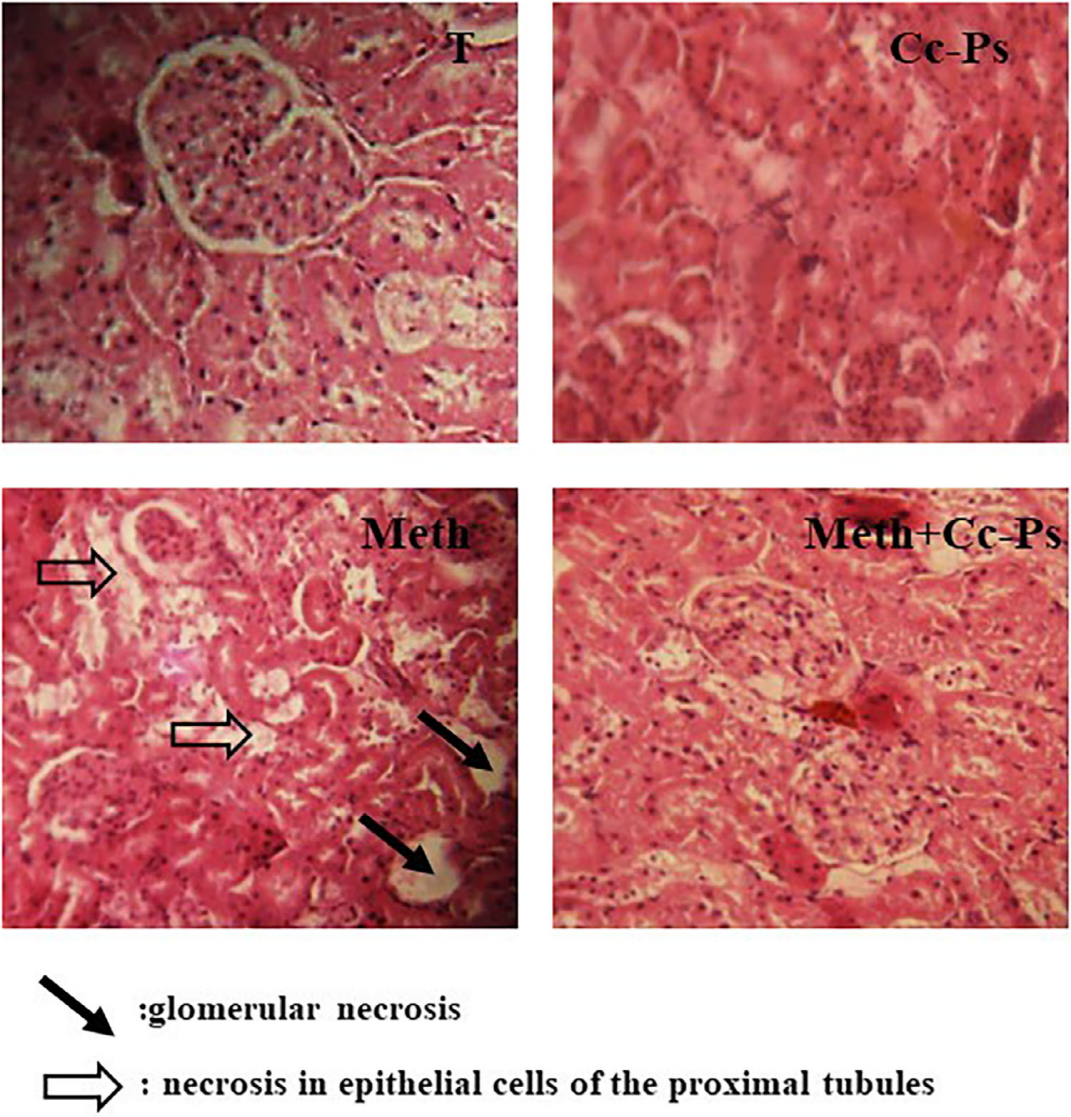
Kidney histopathological changes induced by exposure to MET. Representative photographs showing (T) a control rat liver section, (Cc‐Ps) a liver section from a rat consuming polysaccharide extract from *Cinnamon cassia*, (MET) liver sections from methomyl‐treated rats, and (MET + Cc‐Ps) liver sections from polysaccharide pretreated rats. Hematoxylin/eosin staining; magnification ×200.

In the present investigation, we examined sections of liver and kidneys from different treatments to elucidate any histopathological alterations that may be caused by methomyl treatments. Figures 9 and 10 illustrate the sections of liver and kidneys, where the tested groups are cited.

Figure 9 shows normal histological structure of the liver following the treatments of T (control) and Cc‐Ps (polysaccharide extract), where the central vein (VPC) is surrounded by normal hepatocytes (H). The treatment of methomyl group (MET) demonstrated mild dilatation in the central vein and sinusoids, and there was mild vacuolization associated with degeneration in some individual hepatocytes. These changes were moderately improved in polysaccharide pretreated rats (Cc‐Ps + MET).

As seen in Figure 10, the histopathological changes of kidney were observed after exposure of different groups to treatments in the present study. No histopathological alteration with normal structure of the glomeruli (g) and tubules (t) at the cortex was observed in the groups T and Cc‐Ps, whereas the rats treated only with (MET) showed glomerular and epithelial cells of the proximal tubules. However, the polysaccharide extract of *C. cassia* (Cc‐Ps + MET) slightly attenuated these cytological manifestations.

## 4 Discussion

This study examined the toxic effects of the insecticide MET on various physiological, biochemical, and histopathological parameters in male rats. Additionally, the research explored the potential protective effects of cinnamon polysaccharide extract against the toxicity induced by MET. In toxicity studies, researchers assess various specific biochemical parameters to evaluate the physiological and metabolic functions that impact target organs and any potential tissue damage [25]. Typically, the levels of aspartate aminotransferase (AST) and glucose are measured to assess hepatotoxicity, whereas urea and creatinine levels are evaluated to examine glomerular function [26]. The liver and kidneys are critical organs involved in the detoxification of xenobiotics. Our findings indicate a significant elevation in glucose levels. Supporting this observation, AL‐Shinnawy [27] reported that the administration of methomyl led to a notable increase in glucose levels. This suggests potential mechanisms such as enhanced breakdown of hepatic glycogen, elevated levels of adrenocorticotropic hormone and glucagon, or disruptions in carbohydrate metabolism due to reduced insulin activity [28]. Recent observations indicate a notable rise in AST (aspartate aminotransferase) activity, which serves as a critical biomarker for assessing liver injury and toxicity. According to Xuan et al. [29], transaminases like AST are particularly sensitive indicators of liver damage. Elevated levels of serum AST may suggest obstructive damage to the hepatobiliary system as well as significant disruptions to liver cell function, often linked to exposure to pesticides, as highlighted by Li et al. [30]. Our findings align with those of Akhtar et al. [31], who identified a correlation between increased liver biomarkers and imbalances in oxidative stress and antioxidant capacity.

Nephrotoxicity can be observed through elevated levels of creatinine and urea in the serum of male rats treated with methomyl. According to Mowafy et al. [32], this pesticide leads to an increase in urea levels, likely due to heightened nitrogen retention resulting from impaired renal function attributed to significant defects in glomerular filtration. When renal failure occurs, it is characterized by a rapid and progressive decline in the kidney's ability to filter blood, which is further evident by rising creatinine levels. However, pretreatment with *C. cassia* polysaccharide extract significantly corrects these variations. This is explained by the ability of polysaccharides to regulate glycogen metabolism in the liver, possibly through the Akt/FoxO1 signaling pathway, without altering glucose homeostasis and hormone levels [33]. Several studies clearly support our results, demonstrating the hepatoprotective effect of polysaccharides that can inhibit the massive production of methomyl‐induced cytotoxic radicals that can destroy liver cells.

A study by El Khoury et al. [34] showed that polysaccharides derived from *C. cassia* improve insulin sensitivity in the body, which may explain the mechanism of blood glucose reduction in rats pretreated with polysaccharides extracted from cinnamon bark. Supplementation with *C. cassia* polysaccharides significantly normalized the measured parameters. A recovery of renal parameters was observed with a reduction in serum urea and creatinine levels compared to methomyl‐treated rats. This correction was ensured by the protective effect of polysaccharide extracts from cinnamon bark. These polysaccharides exert a nephron‐protective effect thanks to their strong biological, antioxidant, and antiradical properties [35].

Carbamate pesticides, including MET, can induce oxidative stress in rat tissues, leading to an excessive production of free radicals that cause neurotoxicity [36]. In the existing literature, there are several studies focusing on hepatorenal toxicity and oxidative stress caused by MET in rats [37].

This study illustrates that administering methomyl to rats leads to a significant increase in LPO, as indicated by elevated levels of malondialdehyde (MDA) in the liver and kidneys of treated animals. Research by Shanmugam et al. [38] indicates that LPO contributes to the formation of lipid hydroperoxides in cellular membranes, which can damage membrane structure and inactivate membrane‐bound enzymes. The rise in MDA levels attributed to methomyl may result from the binding of methomyl or its degradation products to polyunsaturated fatty acids, as explained by Gutteridge and Halliwell [39]. This interaction can lead to the excessive production of reactive oxygen species (ROS), as highlighted by Garg et al. [40]. Additionally, Sekiyu et al. [41] reported that liver and kidney tissues have relatively high concentrations of fatty acids, which are particularly susceptible to peroxidation. Consequently, oxygen radicals can react with polyunsaturated fatty acid residues in phospholipids, leading to the generation of harmful products, including proteins that can impair various membrane‐bound enzymes [42].

Methomyl has been found to exert toxic effects primarily through the induction of peroxidative damage to cell membranes in the liver and kidneys. This damage occurs as a result of the inhibition of antioxidant enzymes responsible for scavenging free radicals [37]. Studies involving rats treated with methomyl have shown a decrease in the activities of SOD and GPX in both the liver and kidneys, alongside a significant increase in CAT activity. These findings align with the observations made by El‐Demerdash et al. [43], who found that antioxidant enzymes (SOD, CAT, and GST), GSH, and T‐SHç levels were found to be decreased in mice kidney. The elevated CAT activity in methomyl‐treated rats suggests an adaptive response to the increased levels of free radicals, highlighting a failure of the overall antioxidant defense mechanism that is critical for protecting tissues from oxidative damage [44]. Consequently, oxidative stress, arising from either an increase in free radical production or a decrease in antioxidant levels, can lead to the impairment of biological macromolecules and disrupt metabolic and physiological functions within the organism [45]. Notably, the observed decrease in GPX activity among methomyl‐treated rats is associated with diminished levels of its substrates, including low levels of GSH and elevated peroxide concentrations. Prior research has suggested that such reductions may be attributed to decreased synthesis and/or inactivation of relevant enzymes [46].

Moreover, various studies indicate that the organ damage linked to methomyl exposure is likely related to the accumulation of ROS [47]. Additional findings have connected methomyl‐induced tissue damage with excessive production of free radicals and ROS, resulting in increased LPO, DNA damage, protein degradation, and a depletion of the antioxidant defense system [48].

However, administration of *C. cassia* polysaccharides as a pretreatment for 17 days restored the activity of antioxidant enzymes and significantly reduced TBARS in liver and kidney tissues of pretreated rats. Our extract appears to have a protective effect that inhibits the action of free radicals generated by methomyl‐induced oxidative stress. Similarly, the capacity of polysaccharides and the fact that they have multiple antioxidant and antiradical properties explain their hepato‐ and renal‐protective effects against complications and oxidative damage stimulated by methomyl. Moreover, studies by Xu et al. [49] and Wang et al. [50] showed that polysaccharides exhibit protective activity against LPO and cellular damage.

The antioxidant capacity of polysaccharides contained in cinnamon provides a preventive effect against the harmful toxic effects of methomyl. This effect is reflected in the maintenance of redox balance in liver and kidney cells, despite the prooxidant and cytotoxic effects of methomyl, thus protecting these cells from cytolysis. Polysaccharides are good antioxidants because they scavenge various types of ROS, such as superoxide, hydroxyl radicals, hydrogen peroxide, and nitrogen radicals [51]. Previous studies have reported that polysaccharides show good inhibitory ability against LPO [52] and are also known for their anti‐inflammatory effects, thus providing protection against the harmful effects of pesticides and some environmental pollutants [53]. Furthermore, Hashemzae et al. [54] demonstrated that polysaccharides inhibit ROS formation and reduce nephrotoxicity caused by glyoxal, thus the nephroprotective effect of polysaccharides.

This study delves into the remarkable antioxidant properties of polysaccharides through a series of in vitro assays, including DPPH, FRAP, and ABTS. The findings unveil compelling evidence of significant reducing capabilities, tied to compounds adept at transforming ferricyanide complexes from their oxidized Fe^3+^ state to the more reduced Fe^2+^ state. Remarkably, the extract's reducing power surpassed that of BHT, showcasing its potential as a potent antioxidant and inviting further exploration into its benefits and applications. This effect is primarily linked to the presence of hydroxyl groups in phenolic compounds, which function as electron donors [55]. Additionally, the polysaccharide extracts from *C. cassia* demonstrated substantial free radical scavenging activity, as evidenced by the reduction of stable and free radicals such as DPPH and ABTS. This activity is likely due to the extracts’ ability to donate hydrogen and electrons. Our observations align with those reported by Huihui et al. [56] concerning cinnamon plants, highlighting the significant contribution of polysaccharides to the antioxidant capacity in medicinal plants.

Regarding antibacterial activity, the results of this study showed that the polysaccharide extract exhibited antibacterial activity against *Enterococcus faecalis*, *Escherichia coli*, and *Staphylococcus aureus*. *E. faecalis* unveiled its remarkable prowess with an impressive inhibition zone measuring around 21 mm, a striking testament to its antimicrobial strength. Close on its heels, *S. aureus* showcased its resilience with an inhibition zone of 20.33 mm, whereas *E. coli* displayed its own noteworthy presence at 20 mm, highlighting the fierce competitiveness within this microbial arena. These results are consistent with those of Kačániová et al. [57], who reported that polysaccharides from *C. cassia* did not show the same effective antibacterial activity against both bacterial species. Similar to the results of Maher et al. [58], who indicate that the high resistance observed in Gram‐negative bacteria can be attributed to the intricate structure of their cell wall. Unlike Gram‐positive bacteria, which possess a simpler membrane structure, Gram‐negative bacteria have a more complex cell wall characterized by a double membrane. This unique architectural feature contributes to their enhanced resistance to various external factors.

Histological studies showed that MET (4 mg/kg) was one of the most common liver lesions and changes resulting from methomyl treatment, including cell death of hepatocytes [59]. Rats treated with MET had portal vein obstruction, poorly contoured hepatocytes, and sinusoids [60]. The results of this study are consistent with those of Muthuviveganandavel et al. [61], who observed multiple necrotic areas of hepatocytes infiltrated with mononuclear cells after treatment of rats with carbendazim or carbosulfan. Hepatocyte degeneration and necrosis may be due to vascular changes, especially in the portal vein. Stojisavljević1 et al. [62] showed that exposure to methomyl increases oxidative damage to hepatocytes by causing lipid membrane peroxidation and ultimately leading to apoptosis. Recent studies have highlighted significant damage to kidney structure following exposure to this pesticide. In rats treated with methomyl, notable pathological changes were observed, including degeneration of renal tubules, severe congestion of central vessels, and dilation and congestion of glomeruli. These alterations are attributed to increased LPO resulting from exposure to this toxic substance. Our histological and biochemical results are consistent with those of Chabane et al. [63].

Molecular docking investigations performed against “*D. melanogaster* Acetylcholinesterase” (pdb: 6XYU) show the interesting biological effect of the docked phytomolecules which showed significant binding affinity values in addition to significant intermolecular interactions reflecting the inhibition of the target enzyme, whereas docked MET showed a very low score (high binding energy) and weak interactions (van der Waals and Pi–Alkyl) which show no strong AChE inhibition. MET via these weak interactions, results in cholinergic overstimulation and neuromuscular dysfunction, and may cause various health risks.

Recent studies indicate that polysaccharides from the bark of *C. cassia* offer significant protective effects on vital organs like the liver and kidneys, particularly under oxidative stress caused by the pesticide methomyl. These polysaccharides are effective antioxidants, scavenging harmful free radicals generated by methomyl and helping to preserve cellular function. This highlights the potential therapeutic benefits of *C. cassia*‐derived polysaccharides in combating pesticide‐related damage and promoting organ health.

## Author Contributions

**Nozza Bouzenna**: writing – original draft, methodology. **Mabrouk Horchani**: writing – review and editing. **Sabah Dhibi**: writing – review and editing. **Hafsia Bouzenna**: methodology, conceptualisation. **Fatma Guesmi**: writing – review and editing, formal analysis. **Anouar Feriani**: methodology, formal analysis. **Hichem Ben Jannet**: visualisation, validation. **Sirine Choura**: conceptualization. **Angelo Maria Giuffrè**: software; writing – review and editing; visualization; supervision; project administration. **Mohamed Chamkha**: writing – review and editing, project administration. **Najla Hfaiedh**: supervision. project administration, writing – review and editing, software, validation.

## Funding

The authors have nothing to report.

## Conflicts of Interest

The authors declare no conflicts of interest.

## Data Availability

The data that support the findings of this study are available from the corresponding author upon reasonable request.
